# Implementation and Evaluation of a Community Resource Assessment Process to Identify and Expand Partnerships That Support a Cardiovascular Disease Risk Reduction Program for Uninsured Women

**DOI:** 10.5888/pcd22.240412

**Published:** 2025-05-15

**Authors:** Kristine Zimmermann, Chloe Ford, Leslie Carnahan, Pam Jefferies, Phallisha Curtis, Crystal Magallon, Manorama Khare

**Affiliations:** 1 University of Illinois College of Medicine Rockford; 2 University of Illinois Cancer Center, Chicago; 3 School of Public Health, University of Illinois Chicago; 4 Illinois Department of Public Health, Springfield

## Abstract

Low-income, uninsured women are disproportionately affected by cardiovascular disease (CVD). Facilitating primary and secondary prevention of CVD in this population requires supports beyond clinical and public health agencies. The Illinois WISEWOMAN Program (IWP) provides CVD prevention, screening, and management for women who lack health insurance in Illinois, including the use of referral systems to link clients to needed services. To support these efforts, the 7 local agencies implementing IWP developed linkages to facilitate participant referrals for health promotion, clinical services, and programs addressing social needs. The IWP evaluation team implemented a community resource assessment (CRA) process to describe partnerships and facilitate partnership expansion. To conduct the process, we developed a CRA template, which IWP agencies completed in 2019, 2021, and 2023 to describe community organizations that offer IWP-related resources, their relationships with these organizations, and plans to develop or maintain relationships. We tabulated data on the number of partnerships at each agency and across resource types, mapped findings, and compared data across years. We also consolidated and shared strategies for partnership development. The number of partnerships increased from 2019 (N = 179) to 2021 (N = 225), then decreased slightly to 214 by 2023. In 2023, several IWP agencies reported that partners had discontinued services due to the COVID-19 pandemic. The CRA process provides a formal structure for public health agencies to document their partners and gaps and plan for new partnerships. Additionally, the CRA process contributes to understanding the diverse contexts in which public health programs are offered and how external factors, such as COVID-19, can indirectly affect the availability of community resources.

**Summary**

**What is already known on this topic?**

Difficulties in accessing health screening and health-promoting resources can contribute to disparities in health outcomes among populations vulnerable to poor health outcomes. The National WISEWOMAN program prioritizes linkages with clinical and community partners to support access and use of services, ultimately improving cardiovascular outcomes.

**What is added by this report?**

To facilitate linkages with clinical and community partners, we developed and implemented a structured community resource assessment process to assist public health agencies in documenting their partners, partnership gaps, and plans for developing and maintaining partnerships.

**What are the implications for public health practice?**

Our community resource assessment process facilitated implementing agencies’ awareness of available referral partners and planning for partnership expansion, provided data to evaluate partnership changes over time, and elicited a better understanding of the contexts in which WISEWOMAN was offered.

## Introduction

Cardiovascular disease (CVD), the leading cause of death among US women (1), can be delayed or prevented through screening, behavior modification, and management of clinical risk factors like hypertension and diabetes (2). Low-income, uninsured women without access to preventive health services may be unaware of their CVD risk and lack resources to support risk-reduction behaviors (3). To address this need, in 1993, the Centers for Disease Control and Prevention (CDC) developed the Well-Integrated Screening and Evaluation for WOMen Across the Nation (WISEWOMAN) program. This program addresses CVD risk among uninsured women aged 40 to 64 years through screening, risk reduction counseling, and referrals to health care for identified needs and evidence-based preventive services such as the Diabetes Prevention Program (DPP) and community-based weight-loss programs (4). WISEWOMAN has demonstrated effectiveness in improving women’s health outcomes, including timely clinical follow-up after elevated blood pressure screening, smoking cessation, and reduced blood pressure in participants with hypertension (5–7).

During the 2018–2023 WISEWOMAN program cycle, CDC continued to emphasize clinical and community linkages to improve coordination among partners, including through bidirectional referrals, and to facilitate access to opportunities to promote healthy behavior among WISEWOMAN clients (8).

In Illinois, WISEWOMAN is facilitated by the Illinois Department of Public Health and implemented by 2 federally qualified health centers (FQHCs) and 5 local health departments located in 4 metropolitan and 2 nonmetropolitan counties (9). Together, the IWP agencies reached 3,191 unique participants over 5 years. Participation varied widely: a nonmetropolitan-based local health department serving 1 county had the fewest participants (N = 50), while a metropolitan-based FQHC had the most participants (N = 2,304).

IWP agencies have a history of partnering with local organizations to refer participants to evidence-based programs, clinical services, and programs that address social determinants of health. Often, these partnerships increase community awareness about IWP, which can support recruitment. Interagency collaboration can be beneficial for public health practice, but relationships can be affected by organizational capacity, communication between agencies, and use of referral resources (10–13).

## Purpose and Objectives

In the national WISEWOMAN program, state-based programs were charged with linking community and clinical resources for women at risk for CVD to identify and refer participants to relevant services (short-term outcomes), which contribute to increased participation in healthy behavior support services and behavior change to reduce CVD risk (intermediate outcomes), and ultimately contribute to blood pressure control among women with hypertension (long-term outcome) (8). To support IWP agencies in this component of WISEWOMAN and enhance our process evaluation of short-term and intermediate objectives, the IWP evaluation team developed a community resource assessment (CRA) process. This process allowed IWP agencies to identify partners and potential partners and assisted them with strengthening and expanding partnerships. Prior research described the role of CRAs to identify partnerships and partnership gaps, support health-related interventions, or initiate the process of addressing health disparities (14–16). In our evaluation, each IWP agency completed a CRA in 2019, 2021, and 2023 (program years 1, 3, and 5). The CRA process was deemed not human subjects research by the University of Illinois College of Medicine Rockford Institutional Review Board.

Through the CRA process, we sought to understand the number and breadth of existing community partnerships by agency, partnership gaps, and the extent to which partnerships changed during the program, hypothesizing that the number and breadth of partnerships would expand over 5 years. Beginning in Year 3 (2021), we modified the CRA template to understand how agency partnerships were affected by the COVID-19 pandemic, hypothesizing that the addition of COVID-related resources would further expand partnerships.

## Intervention Approach

In WISEWOMAN, CDC required partnerships to refer clients to healthy behavior support services (eg, DPP, weight-loss programs); however, we recognized that immediate needs, including medical, socioeconomic, and family concerns, may need to be addressed before engagement in CVD-risk–management behaviors (17).

To create the CRA template, we used previously developed community asset mapping concepts and materials, with a focus on organizations, services, and businesses providing resources relevant to IWP and IWP clients (18,19). The CRA template had 7 categories for listing local resources: healthy eating, healthy behavior support services (eg, DPP, weight-loss programs), physical activity, tobacco cessation, miscellaneous health (eg, prescription assistance, mental health), other miscellaneous resources (eg, housing assistance, legal aid, other resources related to social needs), and space to list organizational names within each category.

Beginning in Year 1, IWP staff members from each agency filled in the template, documenting their relationships with external organizations as established, developing, or not partners. “Established partners” were those in which the IWP agency reported engaging in bidirectional client referrals; “developing partners” involved IWP referrals to the partner organization but no reciprocal referrals; “nonreferral organizations” described local organizations or programs within organizations that the IWP agency was not working with. IWP agencies created outreach plans for each developing and nonreferral organization to develop or enhance partnerships. In years 3 and 5, the evaluation team prepopulated templates for each agency and asked them to provide updates on any changes listed in the previous assessment. COVID-19 resources were added to the template in years 3 and 5.

After completing the CRA, the evaluation team presented results to agencies, including the range of partnerships and recommendations for partner outreach and engagement to support shared learning for outreach engagement. Additionally, the evaluation team created visual maps of partnerships, which allowed the Illinois Department of Health and IWP agencies to identify partnerships, partnership gaps, and partnership changes across 5 years.

The CRA process assumes that assets and resources exist across all communities and that creating organizational linkages is beneficial for IWP implementation and outcomes. The process also assumes that community organizations and their services are dynamic, and it is critical to reassess partnerships periodically and actively engage with collaborators to maintain partnerships (18,19).

## Evaluation Methods

The purpose of the CRA process evaluation was to understand resources and resource gaps within and across IWP agencies and provide contextual information for quantitative program data (eg, inadequate local weight-loss programs can inhibit client participation). We created the CRA template as a Microsoft Word document that could be modified (eg, rows added) by IWP agencies as needed. We emailed the document to all IWP agencies and asked them to self-report their partnerships as well as organizations with which they did not have current partnerships. All 7 IWP agencies completed a CRA and returned them via email; 1 agency serving 5 counties completed a CRA for each.

To evaluate the CRA process, the evaluation team tabulated data on nonreferral organizations, developing partners, and established partners by type of resource for each agency and across all agencies. We also consolidated information on outreach strategies described by IWP agencies. During Year 5, we used NodeXL software (Social Media Research Foundation) and the CRA data to create community resource maps for each IWP agency. We created agency-specific images for each CRA year, which showed the changes in partnerships during the 5-year program. NodeXL is a network analysis tool that allows the visualization of complex social networks.

## Results

**Figure 1:**
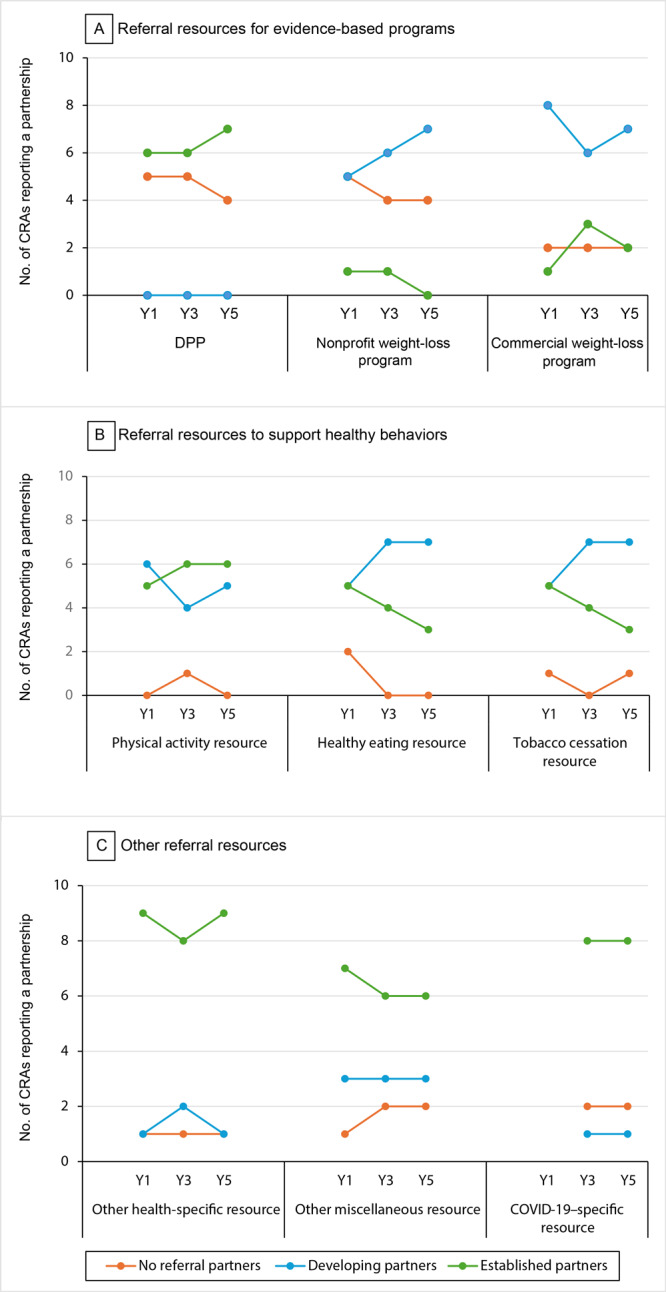
Number of agencies reporting at least 1 type of partnership (none, developing, established) in each type of resource. Data obtained through 11 community resources assessments by 7 Illinois WISEWOMAN Programs in 2019 (Year 1), 2021 (Year 3), and 2023 (Year 5). COVID-19–specific resources were reported in years 3 and 5 only. Abbreviations: DPP, Diabetes Prevention Program; WISEWOMAN, Well-Integrated Screening and Evaluation for WOMen Across the Nation.

Across the 11 CRAs created by 5 local health departments and 2 FQHCs, the number of agencies that had at least 1 type of partnership in each category of resource varied. Most agencies (n = 8 or 9) reported having established a partnership with at least 1 “other health-specific” organization across all time points, and most agencies (n = 8) reported having established a relationship with at least 1 COVID-19–specific resource in years 3 and 5 (Figure 1). Slightly more than half of agencies reported having at least 1 established partnership with a DPP (n = 6 or 7), a physical activity resource (n = 5 or 6), and an “other miscellaneous” resource (n = 6 or 7) at all time points. Most agencies reported having established or developing partnerships with at least 1 healthy eating program across all time points (n = 10 or 11). The number of agencies with an established relationship with at least 1 type of weight-loss program or tobacco cessation resource decreased or remained low during the 5-year study period: for weight-loss programs, from 2 relationships in Year 1 to 2 relationships in Year 5; for tobacco cessation, from 5 relationships in Year 1 to 3 relationships in Year 5.

**Table:** 

Type of resource and year of study	Type of partnership reported, no. of agencies reporting		
	No referral partners	Developing partners	Established partners
DPP			
Y1	5	0	6
Y3	5	0	6
Y5	4	0	7
Nonprofit weight-loss program			
Y1	5	5	1
Y3	4	6	1
Y5	4	7	0
Commercial weight-loss program			
Y1	2	8	1
Y3	2	6	3
Y5	2	7	2
Physical activity resource			
Y1	0	6	5
Y3	1	4	6
Y5	0	5	6
Healthy eating resource			
Y1	2	5	5
Y3	0	7	4
Y5	0	7	3
Tobacco cessation resource			
Y1	1	5	5
Y3	0	7	4
Y5	1	7	3
Other health-specific resource			
Y1	1	1	9
Y3	1	2	8
Y5	1	1	9
Other miscellaneous resource			
Y1	1	3	7
Y3	2	3	6
Y5	2	3	6
COVID-19–specific resource			
Y1	0	0	0
Y3	2	1	8
Y5	2	1	8

Across agencies, the total number of partnerships and the number of developing partnerships and established partnerships increased from Year 1 to Year 3 and was relatively stable from Year 3 to Year 5. Specifically, in Year 1, agencies reported a total of 179 partnerships (n = 113 developing, n = 66 established). In Year 3, the total number of partnerships increased to 225 (n = 128 developing, n = 97 established). The number of partnership types, by resource type, were mostly stable from Year 1 to Year 3; the largest increases were the addition of 7 “other” health-specific resources and 37 COVID-19–specific partnerships. In Year 5, the number of agency-reported partnerships decreased to 214 (n = 121 developing, n = 93 established). During the 5-year study period, agencies reported the greatest decreases in developing and/or established partnerships with commercial and nonprofit weight-loss programs (n = 27 in Year 1, n = 20 in Year 5) and physical activity partners (n = 33 in Year 1, n = 29 in Year 5), and increases in DPP programs (n = 12 in Year 1, n = 15 in Year 5), tobacco cessation resources (n = 18 in Year 1, n = 20 in Year 5), and “other” health-specific partnerships (n = 30 in Year 1, n = 36 in Year 5).

**Figure 2:**
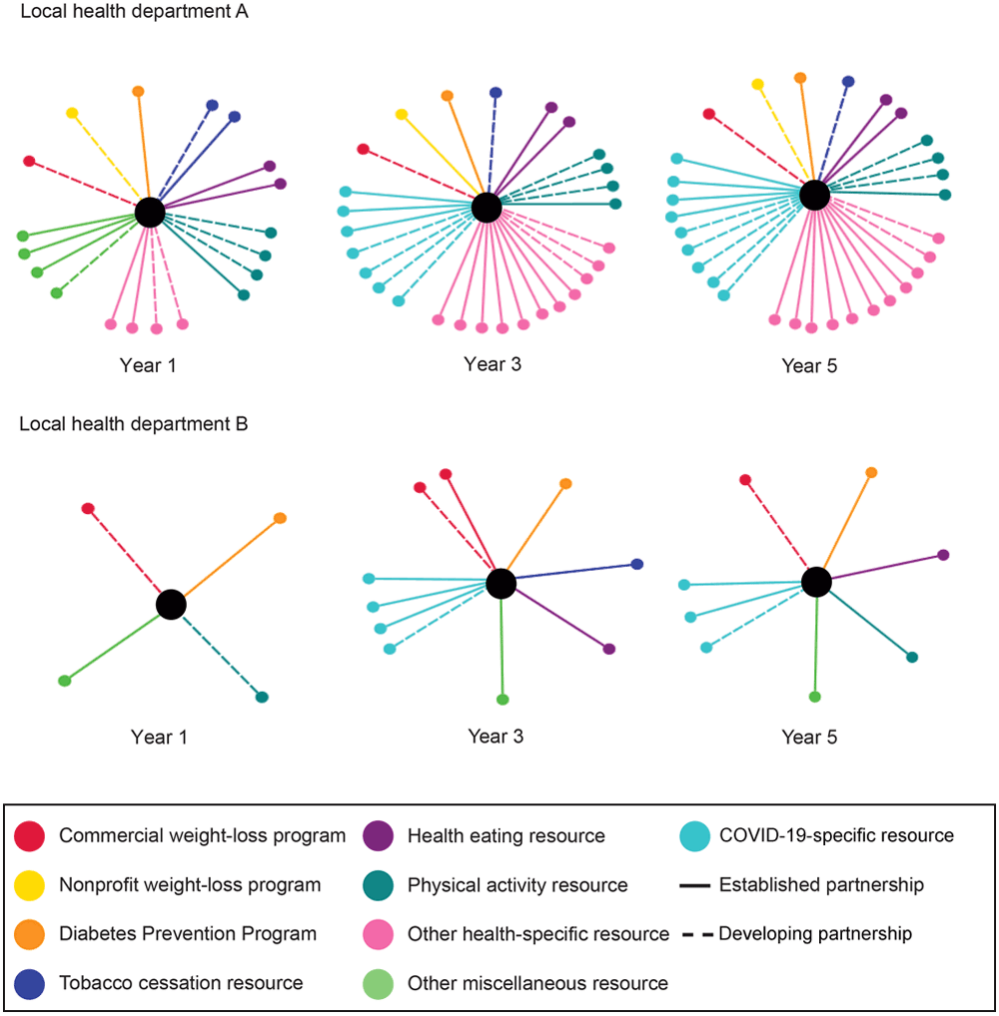
Example of network maps depicting changes in the number of partnership types (established or developing) and 9 resource types at 2 WISEWOMAN program agencies (local health departments A and B) in Illinois during a 5-year cycle (2018–2023).

In the example of network maps depicting changes in partnership type and resource type at 2 IWP agencies (Figure 2), both agencies show changes in partnerships between years 1 and 3 but few changes between years 3 and 5.

While most partnerships were external to IWP agencies, some partnerships were with programs or services within the agency implementing IWP. In Year 1, the number of such internal partnerships ranged from 0 and 2 per agency (total across agencies = 7): mental health services in both FQHCs, DPP programs in 2 LHDs and 1 FQHC, and 2 agency-specific “healthy behavior support services.” In Year 3, internal partnerships ranged from 0 to 3 per agency (total across agencies = 12). Partnerships increased through linkages with internal COVID-19 testing and vaccination sites, and 1 FQHC lost its internal partnerships due to closure of its affiliated hospital. In Year 5, changes in the number of internal partnerships were minimal.

In their outreach plans across all time points, IWP agencies emphasized communication strategies to develop or maintain partnerships, including identifying a point person at potential partner organizations, conducting in-person visits or sending letters to share IWP materials (eg, brochures) and following up by telephone, and working with the agency’s community navigator to develop partnerships. With existing partners, IWP agencies planned regular check-ins, including giving updates about IWP and thanking partners. In Year 5, some IWP agencies reported the COVID-19 pandemic as a barrier to partnership development due to staffing reallocation and social distancing.

In years 3 and 5 updates, IWP agencies reported their level of services offered. For example, in Year 3, IWP agencies reported changes in services due to COVID-19 (eg, in-person services discontinued, virtual services offered; normal operations). In Year 5, IWP agencies reported whether services had returned to pre–COVID-19 operations, continued in a limited capacity, or had been discontinued.

## Implications for Public Health

By mapping referral partnerships used in the IWP program, we assisted IWP agencies in documenting resource adequacy, gaps, and plans for expanding partnerships to better support their IWP clients. The national WISEWOMAN program aims to engage women in evidence- and community-based programs to reduce CVD risk by supporting behavior change through healthy eating, physical activity, and weight loss (4,8). However, the lack of partnerships with such resources in communities offering WISEWOMAN inhibits such engagement. While most IWP agencies had established partnerships with health-related organizations, including COVID-19–specific resources, established referral partnerships to support engagement in evidence-based healthy lifestyle programs and health behavior opportunities were often limited.

While the CRA process was intended to support partnership development, agencies’ ability to develop and maintain partnerships was unexpectedly affected by the COVID-19 pandemic. In 2020, during statewide shutdown in Illinois, IWP agencies reported serving as information hubs on accessing community resources, including basic needs, COVID-19 testing, and COVID-19 vaccinations. The pandemic also changed the community resource landscape. For economic and other reasons, some in-person programs, including weight-loss and community-based fitness centers, were discontinued or permanently closed. By mapping resource changes over time, we visually demonstrated how the pandemic affected public health resources within communities and, more broadly, how a public health emergency can indirectly affect other community services. For IWP, the decrease in referral partnerships for weight loss and physical activity contributed to fewer opportunities for IWP clients.

One advantage of the CRA process was the opportunity for IWP agencies to identify community resources beyond those directly related to WISEWOMAN services (eg, clinical services to address health conditions identified through WISEWOMAN, weight-loss programs, tobacco-cessation resources). For example, people with or at risk for chronic disease are more likely to have social needs that can affect their engagement in health-related behaviors (20). Thus, uninsured, low-income women, such as those served by WISEWOMAN, must meet their most urgent needs before they can focus on CVD-risk–reduction activities (17). However, local health departments and FQHCs may be ill-equipped to address the various needs of clients. In our analysis, the number of agencies with at least 1 established referral partner in the “other miscellaneous resource” category decreased over time, and those with no referral partners increased. Because sustained partnerships with referral resources to address social needs are essential to reducing the health disparities of populations vulnerable to disease, aligning community resources and supporting cross-sectoral collaboration beyond typical clinical and public health organizations is critical (17).

We plan to continue the CRA process with several enhancements. In the current WISEWOMAN program cycle (2023–2028), participants are assessed for and receive referrals for social needs, and our new CRA template asks agencies to denote social determinant of health–related resources (eg, childcare, food access, transportation). Tracking participant referrals and their use of services and combining participant and CRA data will help us to understand community-specific social needs, whether agency-specific partnerships are adequate to meet these needs, and whether resource gaps must be addressed. We also plan to use social network analysis to evaluate the reciprocity of IWP-related partnerships and interconnectedness among IWP partners, strengthen partnerships, improve information- and resource-sharing across organizations, and improve long-term health outcomes (14). These enhancements will allow us to identify areas needing additional technical assistance to better support IWP clients and other people with health and social needs.

Through a process evaluation that was also an intervention for IWP agencies, we identified partnerships and resource gaps to support agencies in serving their client populations. One limitation of our evaluation was our inability to quantify referrals made to or received from partners. A robust partnership network can ensure that diverse needs of IWP participants are met; however, we are unable to discern whether partnerships were adequate to meet participant needs. Other limitations were the lack of perspectives from partner agencies to validate the types of partnerships and the lack of perspectives from IWP participants, which would be beneficial for attaining in-depth information on the effectiveness of referral partnerships.
